# Editors’ Book Table

**Published:** 1873-04

**Authors:** 


### Editors' Book Table.

[NOTE. — All works reviewed in the columns of the CHICAGO MEDICAL JOURNAL may be found in the extensive stock of W. B. KEEN, COOKE & Co., whose catalogue of Medical Books will be sent to any address upon request.]

BOOKS RECEIVED.

*The Microscope and Microscopical Technology: a Text Book for Physicians and Students.* By Dr. HEINRICH FREY, Professor of Medicine in Zurich, Switzerland. Translated from the German and Edited by GEORGE R. CUTTER, M.D., Clinical Assistant to the New York Eye and Ear Infirmary. Illustrated by 343 Engravings on Wood; and Containing the Price Lists of the Principal Microscope Makers of Europe and America. From the Fourth and last German Edition. New York: William Wood & Co., 27 Great Jones Street. 1872. Pp. 658. Cloth, $6.00.

In the January No. of the JOURNAL is briefly noticed the reception of this book. Since some one of our professional friends borrowed and forgot to return our copy of Beale's "How to Work with the Microscope," our library has mourned over a *hiatus valde deflendus.* There was an "aching void." But all evils have their compensations. In this book of Prof. Frey we find the whole matter "posted up to date." The volume is brought out in elegant style, and, as it should be, is profusely illustrated. The arrangement is satisfactory in every particular, giving first, the Theory of the Microscope, then successively: Apparatus for Measuring and Drawing; the Varieties and Modes of Testing; Use and Methods of Examination; the Preparation of Objects; Fluid Media and Chemical Reagents; Methods of Staining, Injecting, etc.; Mounting and Arrangement of Objects—the eleven concluding chapters being devoted to a careful delineation of the microscopy of the fluids and solids of the human body. A price list of the leading manufacturers follows the index. The more carefully we examine this book, the more thoroughly are we pleased and delighted with it.

The microscope is now the indispensable adjunct of the physician's study, but without instruction in its use, the practitioner will find himself sadly disappointed in using it.

*Club-Foot: Its Causes, Pathology, and Treatment.* Being the Essay to which the Jacksonian Prize for 1864, given by the Royal College of Surgeons, was awarded. By WM. ADAMS, F. R. C. S., Surgeon to the Northern Hospital, and to the National Hospital for the Paralyzed and Insane, etc., etc. With one hundred and six Wood Engravings, and six Lithograph Plates. Second Edition. Philadelphia: Lindsay & Blakiston. 1873. 8vo. Pp. 464. Cloth, $6.00.

This is a superb work and should be in the hands of every surgeon in the country, as it is by far the most complete and instructive treatise on the subject. By the assistance of the numerous and capital illustrations everything is presented in the most perfect manner to the eye and the understanding. The present edition has been carefully revised, many chapters materially added to, especially the one on Preparation of the Tendons, and five new lithograph plates added.

*Contributions to Mental Pathology.* By J. RAY, M.D., Author of "Medical Jurisprudence of Insanity," and Mental Hygiene. Boston: Little, Brown & Company. 1873. Pp. 558.

Most of the present volume has already appeared in print, and the general opinions of Dr. Ray are well known to the professional public. The matter of which it is composed, however, has been thoroughly revised, a scientific arrangement adopted, and a portion of new material added. It is well considered, well digested, and, while it is a very important treatise for the professional man, it will be found well worthy the attention of the intelligent general reader.

*Manual of Chemical Analysis as Applied to the Examination of Medicinal Chemicals. A Guide for the Determination of their Identity and Quality, and for the Detection of Impurities and Adulterations.* For the Use of Pharmaceutists, Physicians, Druggists, and Manufacturing Chemists, and of Pharmaceutical and Medical Students. By FREDERICK HOFFMAN, Ph. D., Pharmaceutist in New York. New York: D. Appleton & Co., 549 and 551 Broadway. 1873. 8vo. Pp. 393.

This is one of the most useful books of the season. The information it contains has been collected from a wide variety of sources inaccessible to most readers. It has been compiled with special reference to the latest Pharmacopœias of the United States, Great Britain and Germany.

The work is divided into two parts, the first of which treats of Operations and Reagents; Reagents; Course of Qualitative Analysis, and Volumetric Analysis; the second, discusses the Medicinal Chemicals and their Preparations in detail. Numerous illustrations are given throughout the volume, and to the whole is appended a series of Tables of great practical service, and a copious Index. We cannot too highly recommend this treatise to our readers. It fills a want we have ourselves long experienced.

*Illustrations of the Influence of the Mind upon the Body, in Health and Disease.* Designed to Elucidate the Action of the Imagination. By DANIEL HACK TUKE, M.D., M.R.C.P., Joint Author of "The Manual of Psychological Medicine," etc., etc.

"There is not a natural action of the body, whether voluntary or involuntary, that may not be influenced by the peculiar state of the mind at the time."—JOHN HUNTER.

Philadelphia: Henry C. Lea. 1873. 8vo. Pp. 415. $5.50.

A book very much needed—worth more, to the thoughtful and judicious physician, than a whole shelf-full of volumes on Materia Medica. Dr. Tuke is already well known, to the reading portion of the profession, through the excellent joint treatise, by Dr. Bucknill and himself, on Insanity. The present essay is more limited in its scope, but brimful of matter indispensable to the professional scholar. The author has collected, from a wide variety of sources, cases, authentic in character, illustrative of the influence of Mind upon the Body. These are supplemented by a great number falling under his own observation, and the whole systematically arranged, in accordance with modern physiological and psychological ideas. The mental influence is shown as causing derangement of sensation, motion, and the organic functions, and its therapeutic importance pointed out. The next effort is to ascertain the channels of this influence, and finally to elucidate, as fully as possible, the exact nature of what is usually designated the Imagination. The book is methodically divided and written in a pleasing style, devoid of abstract and foggy metaphysical speculations, clear and instructive. To employ a phrase we remember to have seen used before, "No library can be complete without it." Most certainly no one can rise from its perusal without a feeling of satisfaction with the book and gratitude to the author. Incidentally, we have noticed, that whilst lying upon the office table, with the other literary, scientific and professional works, latest productions by the press, this one has been the most noticed of all, and solicitations to borrow it most frequent.

*Fistula, Hæmorrhoids, Painful Ulcer, Stricture, Prolapsus and other Diseases of the Rectum: their Diagnosis and Treatment.* By WILLIAM ALLINGHAM, F.R.C.S.E., Surgeon to St. Mark's Hospital for Fistula, etc.; late Surgeon to the Northern Hospital. Second edition, revised and enlarged. Philadelphia: Lindsay & Blakiston. 1873. Pp. 265.

This is a capital addition to the literature of the topics mentioned in the title. It is well written, in good vigorous English, and we fully endorse the opinion of a contemporary, that there is "no book on this special subject that can at all approach Mr. Allingham's in precision, clearness and practical good sense."

*A Treatise on Apoplexy, Cerebral Hemorrhage, Cerebral Embolism, Cerebral Gout, Cerebral Rheumatism, and Epidemic Cerebro-Spinal Meningitis.* By JOHN A. LIDELL, A.M., M.D., Prof. of Anatomy in the National Medical College, Washington, D. C.; formerly Surgeon to Bellevue Hospital, New York, etc., etc. New York: Wm. Wood & Co., 27 Great Jones Street. 1873. 8vo. Pp. 395.

Dr. Lidell elaborates in this volume the ante and post-mortem histories of sixty-two cases of cerebral diseases. Of these, forty-four are original, and besides them many original cases are briefly cited. A special chapter is devoted to *Infantile Apoplexy*, one to the so-called *Pulmonary Apoplexy*, and the closing chapter discusses Cerebro-Spinal Meningitis, which latter disorder he very properly considers a local "inflammation," excited by a specific poison. The author informs us that he started out with the purpose of writing nothing more than an article, or series of articles, for the *American Journal of Medical Sciences*, but the matter soon accumulated to such a bulk that he abandoned that idea and concluded to present it in book form. The result is the volume before us, which is rich in well prepared material for study. Aside from his original descriptions and views, the author cites numerous authorities, which will give the reader an excellent bibliography of the subject. At the present time there is no other treatise on the subjects discussed so fully up to the period. In a subsequent edition we hope to find some minor blemishes corrected, so that its real merit may be more palpable.

*A Treatise on the Theory and Practice of Obstetrics.* By WM. H. BYFORD, A.M., M.D., Professor of Obstetrics and Diseases of Women and Children in the Chicago Medical College, etc., etc. Second edition; thoroughly revised. New York: Wm. Wood & Co., 27 Great Jones Street. 1873. 8vo. Pp. 469. Illustrated.

On the appearance of the first edition of this treatise, we spoke of its general merits in commendatory terms, and foretold its probable popularity. The speedy exhaustion of that edition, and the appearance of the present volume so soon, prove our words correct. Our eminent townsman may well be gratified at the success achieved. Some of our readers are aware of a sharp attack made upon the work by a "hot-livered grammarian," in an eastern magazine, and it is to be confessed the signs of haste in preparation (as must almost of necessity be the case when so busy a man as Prof. Byford writes) were abundantly obvious in that edition. But in the volume before us these blemishes have disappeared, and it can safely challenge the trivial verbal reprobation of the eastern critic. Of its claims as a work of reference and standard authority there can be no room for doubt.

*Santo Domingo, Past and Present: With a Glance at Hayti.* By SAMUEL HAZARD, Author of Cuba with Pen and Pencil. Maps and Numerous Illustrations. New York: Harper & Brothers, Publishers, Franklin Square. 1873.

Mr. Hazard visited the island at the same time with the United States Commission. His description is given in an easy reportorial style, which makes reading also easy. Perusal of it at odd hours, in the terribly cold days of the last month, has almost converted us to annexationists. The variety and perfection of climate embraced in the small space of this one island ought to make it exceedingly valuable as a general sanitarium for physical invalids, as well as the political ones who seek its possession.

PAMPHLETS, ETC.

*The Half-Yearly Abstract of the Medical Sciences.* Being a Digest of British and Continental Medicine, and of the Progress of Medicine and the Collateral Sciences. Edited by WILLIAM DOMETT STONE, M.D., F.R.C.S. (Exam.) Vol. LVI, January, 1873. Philadelphia: Henry C. Lea. 1873. $2.50 per annum, in advance. Single volumes, $1.50. Pp. 296.

*Half-Yearly Compendium of Medical Science.* Part XI. Philadelphia: S. W. Butler & Co., 115 South Seventeenth Street. Pp. 280. $3.00 per annum. Single numbers, $2.00. For the first ten numbers, $12.00.

*Report of the Pennsylvania Hospital for the Insane, for the Year* 1872. By THOMAS S. KIRKBRIDE, M.D., Physician and Chief Superintendent. Published by order of the Board of Managers. Philadelphia: 1873.

*Free Parks and Camping Grounds:* Or, Sanitariums for the Sick and Debilitated Children of Large Cities during the Summer Months. By J. M. TONER, M.D., Washington, D. C.

*Infant Mortality.* By H. C. HAND, M.D., St. Paul, Minn., Ed. N. W. Medical and Surgical Journal.

*The New Operation for Coloring Corneal Opacities.* By R. J. LEVIS, M.D., Surgeon to the Pennsylvania Hospital, and to the Wills Ophthalmic Hospital.

*The Cell.* (Reprinted from "The Lens.") Pp. 16. By I. N. DANFORTH, M.D., Pathologist to St. Luke's Hospital, etc.

*Ryan's Philosophy of Marriage.* In its Social, Moral and Physical Relations; with an Account of the Diseases of the Genito-Urinary Organs, etc. By MICHAEL RYAN, M.D., Member of the Royal College of Physicians. 12mo. $1.00.

*Walker on Intermarriage.* Or the Mode in which, and the Causes why, Beauty, Health and Intellect result from certain Unions, and Deformity, Disease and Insanity from others. With Illustrations. By ALEXANDER WALKER, Author of "Woman," "Beauty," etc., etc. 12mo. $1.50.

*A Report to the Board of Health of the City of Chicago.* On the necessity of an extension of the sewerage of the city. By JOHN H. RAUCH, M.D., Sanitary Superintendent. Published by order of the Board, Chicago. 1873. Pp. 22.

*City of Des Moines, Iowa. Business Opportunity and Desirable Location.*

*McKesson & Roberts' New York Prices Current*, of Drugs, Druggists' Articles, Chemical and Pharmaceutical Preparations, Proprietary Medicines and Perfumery, Sponges, Corks, Dyes, Paints, etc., etc. Pp. 160.

An elaborately and elegantly gotten up catalogue, profusely illustrated, and from the beautiful vignette serving as frontispiece, sent out by a fine looking quartette of gentlemen. The fifth partner probably has not yet earned his spurs.

